# Notes on *Brachymenium* in Guyana with a new species from Mt. Ayanganna

**DOI:** 10.3897/phytokeys.154.39105

**Published:** 2020-08-03

**Authors:** Harold Robinson, G. Karen Golinski

**Affiliations:** 1 Department of Botany, MRC 166, Museum of Natural History, P.O. Box 37012, Smithsonian Institution, Washington, DC, USA Smithsonian Institution Washington United States of America; 2 Department of Botany, The University of British Columbia, Room 3200 – 6270 University Boulevard, Vancouver, BC, Canada The University of British Columbia Vancouver Canada

**Keywords:** African relationship, Bryaceae, peristome, rostrate operculum

## Abstract

A relative of the African species described by Brotherus as *Bryumperspinidens*, has been discovered in Guyana with erect capsules and a short inner peristome. The Guyana material is recognized as a new species, and both species are placed in the genus *Brachymenium*. The characteristics that distinquish the genus are discussed with reference to the Guyana specimens of *Brachymeniumspeciosum*.

## Introduction

Study of bryophyte collections obtained during the Smithsonian Biological Diversity of the Guianas project, has revealed a number of interesting species. Among these are two two collections of a bryaceous moss with capsules identifiable as a *Brachymenium* Schwaegr., Spec. Musc. Suppl. 2(1): 131. 1824, with a leaf that superficially matches the illustration of *Bryumperspinidens* Broth. in the Brotherus treatments in the two editions (1904 and 1925) of Engler and Prantl. The only problems were that the Brotherus illustration was of an African species named as a *Bryum*. The spiniform teeth of the leaf margins were nevertheless similar, and a relationship seemed to be involved. As for the generic placement, the Brotherus (1897) species was described from sterile material so that the placement in *Bryum* Hedw. lacked any real evidence. The relationship of Guyana Highland species to African species fits a pattern noted by Robinson (1965). In addition, there is ample material from Guyana of another species of *Brachymenium*, *B.speciosum* that is newly discussed and illustrated.

## Methods

Specimens in this study were obtained during the Smithsonian Biological Diversity of the Guianas Program conducted over a period of years from 1985 to 2014 (Kelloff et al. 2019). The particular specimens of the new species involved in this study were collected during a separately funded trip conducted by M.D. Clark in 2001 that collected on Mt. Ayanganna. The bryophytes were deposited at the US National Herbarium awaiting identification. They have been in storage since that time.

A note with the specimens indicates that when they arrived in the US they were irradiated during the Anthrax scare of 2001.

## Results

The South American material includes one species that seems to be distinct from others from the Western Hemisphere (see for example Allen 2002) and from the related African species.

### Brachymenium ayangannensis

Taxon classificationPlantaeBryalesBryaceae

H.Rob. & G.K.Golinski sp. nov.

559AD855-EF85-57AE-9984-DA2994EE2C2F

Figure 1

#### Type.

Guyana. Region: Potaro–Siparuni. Mt. Ayanganna, east face, plateau above second of four escarpments. 1380 m, 05°22.550'N, 059°58.350'W. Scrub forest on sandstone and peat, with *Clusia*, *Pagamea* and *Sphagnum*. Epiphyte; sporophytes green. 17 June 2001. *H.D. Clarke 9299*, with *R. Williams*, *C. Perry*, *E. Tripp & J. Kelly* (US).

**Figure 1.**
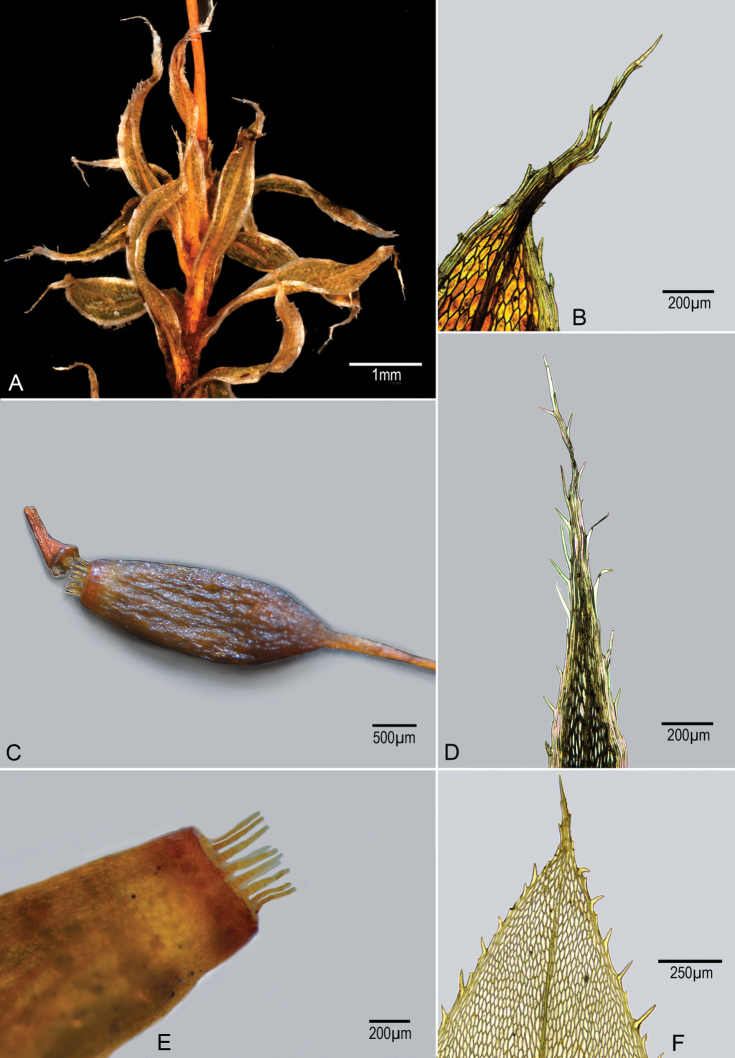
*Brachymeniumayangannensis* H.Rob. & G.K.Golinski. **A** Leafy stem showing distorted leaves **B** distal part of leaves, showing well-developed pale margin and spiniform teeth on both margin and long-acuminate tip **C** remoistened erect capsule showing partially detached short-rostrate operculum **D***B.perspinidens* (Broth.) H.Rob. & Golinski, distal part of leaf of holotype (from H) **E** tip of capsule mounted in Hoyer’s solution showing short exostome and lack of well-developed endostome. The photographs were taken using a Leica DM4B Compound microscope, using a 5× objective.

#### Description.

Stems up to 3 cm tall, leaves not closely spaced, rather firm in structure but contorted when dry and resistant to wetting. Costa percurrent into a long slender acumination, median cells narrowly oval, with firm walls showing slight porosity, mostly 80–100 μm long and ca. 30 μm wide, without shorter quadrate cells at base, margin with numerous rows of linear pale cells forming a strong border, border with numerous cells projecting as spiniform teeth, such spiniform teeth extending onto apical acumination. Synoicous? Seta pale yellowish-red, ca. 17 mm long, smooth. Capsules erect, ca. 2 mm long, with short hypophysis, operculum short-rostrate, higher than wide. Outer peristome teeth reddish, rudimentary, ca. 80 μm long, inner peristome a low pale membrane ca. 70 μm without projecting segments or cilia. Calyptra not seen. Spores ca. 10 μm in size.

#### Additional material.

Guyana. Region: Potaro–Siparuni. Mt. Ayanganna, east face, area near camp at base of fourth of four escarpments. Elev. 1545 m, 05°23.083'N, 059°58.550'W. Dense forest on sandstone and peat, with *Euterpe*, *Clusia*, and *Brocchinia*. Sporophytes green. On tree limb. *H.D. Clarke 9551* with *R. Williams*, *C. Perry*, *E. Tripp & J. Kelly* (US).

The peristome teeth of the new species have proven extremely fragile, possibly because of the radiation treatment.

The spiniform teeth of the leaf margin are distinctive, but the manner in which they occur on the acuminate apical extension is reminiscent of the illustration by Brotherus (1904: 557 fig. C; 1925: 367, figs C, D). This illustration has led to the comparison, but it proves to be somewhat inaccurate compared to the more recent illustration made from the type by Ochi (1972)

The African species is well illustrated by Ochi (1972), but the type from Helsinki has been borrowed not because of doubts of relationship so much as to insure that the two species are not the same. The principle difference is the absence of spinose teeth extending on to the apical acumination of the leaf. Nevertheless, there is no doubt the two are close, and the African species was placed in Bryum only because there was no sporophyte to indicate otherwise. A important point derived from the Ochi study is that none of the species in typical *Bryum* have spinose marginal teeth, all with such teeth are in what is now in the *Brachymenium*, *Rhodobryum* relationship. On the basis of the evident relationship between the African and Guyana species, the following transfer of the African species is provided.

### Brachymenium perspinidens

Taxon classificationPlantaeBryalesBryaceae

(Broth.) H.Rob. & G.K.Golinski comb. nov.

673E4191-B2F7-52F6-B9C3-CE439A8FCF10

Figure 1D

Bryum perspinidens Broth., Bot. Jahrb. Syst. 24: 246. 1897. Britische Ostafrika, Seengebiet: Ru– Nssóro, 3300–3600 m (Uganda: Ruwenzori, heather forest 10–12000’), *Scott Elliot 266*, Sterile. Rhodobryumperspinidens (Broth.) Pócs, in Bizot & Pócs, Acta Bot. Acad. Sci. Hungaricae 25: 257. 1979 [1980]. With record of species from Tanzania, also sterile. Ochi (1972) indicated the species was rather an oddity in BryumHedw.subgenusRhodobryum Schimp. in which he placed it.

#### Notes.

Placement of the new species in *Brachymenium* is based on the capsules being erect with an inner peristome being a low membrane lacking segments or cilia, the traditional distinctions of the genus. Recent DNA studies (Pedersen et al. 2003, Pedersen and Hedenas 2005; Cox and Hedderson 2003) indicate that species that have been placed in the genus *Brachymenium* are mostly in basal branches of the Bryaceae while *Bryum* species are more derived. According to such studies, the genus *Brachymenium* is more entangled phyletically with the genus *Rhodobryum* (Schimp.) Hampe, Linnaea 38: 663. 1874, a later established genus and *Osculatia* De Not., Mem. Reale Accad. Sci. Torino, ser 2, 18: 445. 1859 (Robinson 1965; Ochyra et al. 2018). A survey of the illustrations in Brotherus (1904, 1925) shows an additional trend in *Brachymenium* that is lacking in typical *Bryum*, conical to rostrate opercula such is seen in the new species. In fact, within the present definition of *Brachymenium*, fully rostrate opercula occur in another species recently collected in Guyana.

### Brachymenium speciosum

Taxon classificationPlantaeBryalesBryaceae

(Hook. & Wils.) Steere.

17399882-F918-5D65-968F-86F1F8CCAEAA

Figure 2

#### Notes.

The latter species has been collected on a mountain near Ayanganna as indicated below.

Mt. Wokomung, Little Ayanganna, upper slopes of highest point of Mount Wokomunga massif. 5°5'8"N, 59°50'32"W. elev. 1525 m. Tepui scrub forest on sandstone and peat, with *Schefflera*, *Clusia* and *Guadua*. 5 July 2003, *H.D. Clarke 10550*, with *R. Williams*, *C. Perry*, *J. Kelly*, *D. Gittens*, *S. Stern*; Guiana. Mt. Wokomung, Little Ayanganna, upper slopes of highest point of Mount Wokomung massif. 5°5'8"N, 59°50'32"W. elev. 1525 m. Tepui scrub forest on sandstone and peat, with *Schefflera*, *Clusia* and *Guadua*, elev. 1525 m. 5 July 2003, *H.D. Clarke 10575*, with *R. Williams*, *C. Perry*, *J. Kelly*, *D. Gittens*, *S. Stern.* Guiana. Mt. Wokomung, Little Ayanganna, upper slopes of highest point of Mount Wokomung massif. 5°4'53.1"N, 59°50'26.1"W. elev. 1525 m. Tepui bog on sandstone and peat, with *Brocchinia*, *Bonnetia* and *Rapatea*, elev. 1660 m. 6 July 2003, *H.D. Clarke 10576*, with *R. Williams*, *C. Perry*, *J. Kelly*, *D. Gittens*, *S. Stern.* Mt. Wokomung, area above third of four escarpments, 1 km NE of Mt. Wokomung, 5°4'30"N, 59°51'15"W. elev. 1490 m. dense forest on laterite, with *Clusia*, *Euterpe* and *Licania*, elev. 1490 m. 8 July 2003, *H.D. Clarke 10802*, with *R. Williams*, *C. Perry*, *J. Kelly*, *D. Gittens*, *S. Stern.* The material shows the additional feature of the species, the multistratose leaf margin with teeth on the margin and upper and lower surfaces. The species is otherwise reported from Suriname, Ecuador, and supposedly described from Jamaica (Maracaibo, Venezuela?)(Allen 2013; Steere 1948).

**Figure 2.**
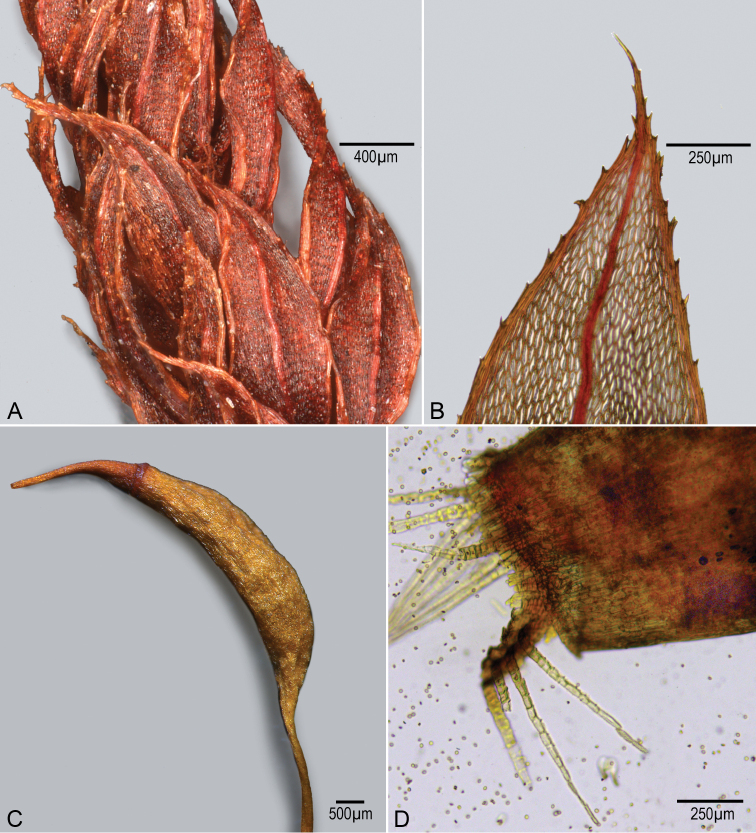
*Brachymeniumspeciosum* (Hook. & Wils.) Steere. **A** Part of leafy stem showing distorted leaves **B** tip of leaf showing thickened margin with teeth on margin and upper and lower surfaces, some of these appearing as double teeth **C** capsule showing rostrate operculum **D** peristome teeth mounted in Hoyer’s solution, showing elongate exostome teeth erect on one half and reflexed on other half, the latter showing endostome lacking cilia and segments. The photographs were taken using a Leica DM4B Compound microscope, 5× objective.

## Supplementary Material

XML Treatment for Brachymenium ayangannensis

XML Treatment for Brachymenium perspinidens

XML Treatment for Brachymenium speciosum
